# Effect of forced swimming stress on count, motility and fertilization capacity of the sperm in adult rats

**DOI:** 10.4103/0974-1208.57226

**Published:** 2009

**Authors:** Ghasem Saki, Fakher Rahim, Karim Alizadeh

**Affiliations:** 1 Department of Laboratory of Cell Culture, Anatomical Sciences, Faculty of Medicine; 2 Physiology Research Center, Joundishapour University; 3 Apadana Clinical Research Center, Private Hospital of Apadana, Ahwaz, Iran

**Keywords:** Fertilization capacity, *in vitro* fertilization, rat, sperm motility, stress

## Abstract

**AIMS:**

The purpose of this study was to determine whether 50 days of forced swimming stress applied to adult male rats affects count, motility and fertilization capacity of sperm.

**SETTINGS AND DESIGN:**

It is a prospective study designed *in vitro*.

**MATERIALS AND METHODS:**

A total 30 adult male wistar rats were used in this study. All rats were divided into two equal groups (n = 15): (1) control group and (2) experimental group. Animals of the experimental group were submitted to force swimming stress for 3 min in water at 32°C daily for 50 days. Then, all male rats were sacrificed, the right epididymides were removed and sperm concentration and motility were determined. The sperm suspension was added to the ova. Fertilization capacity was assessed by counting two-cell embryos 24-26 h after completion of fertilization *in vitro*.

**STATISTICAL ANALYSIS USED:**

Data are reported as mean ± SD and percentage. The difference between the control and experimental groups was determined by the unpaired *t*-test.

**RESULTS:**

The mean and standard deviation of sperm concentration in the control and experimental groups were 60.8 ± 9.3 10^6^/ml and 20.4 ± 5.3 10^6^/ml, respectively. There was a statistical difference of *P* < 0.05 between the two groups in terms of sperm concentration. The percentage of motility in the experimental group was significantly different (*P* < 0.05). The same results were obtained in case of fertility (*P* < 0.05). Stress caused by forced swimming was observed by a significant increase in the latency of the pain response in the hot-plate test (*P* < 0.05).

**CONCLUSIONS:**

These results suggest that forced swimming stress in time course equal or more than spermatogenesis period, i.e. 48-50 days in the rat will be significantly effective to reduce the number and motility of sperms as well as the fertilization capacity.

## INTRODUCTION

Infertility is the inability of a couple to become pregnant (regardless of cause) after 1 year of unprotected sexual intercourse. About 10% of the couples in the United States are affected by infertility.[1] Both men and women can be infertile. According to the American Society for Reproductive Medicine, 1/3 of the infertile subjects is infertile female, 1/3 is linked to male infertility and the remaining 1/3 is due to a combination of factors from both partners. In approximately 20% of the couples causes could not be determined.[1] Psychological stress has been implicated as one of the major causes of idiopathic infertility in both men and women.[2–7]

Many studies have investigated psychological causes that affect male factor infertility,[8–14] but it remains difficult to tease out stress as a cause or consequence of infertility. A variety of stress factors such as microorganisms, hyperthermia and exposure to heavy metals inhibit male reproductive functions and spermatogenesis.[15] In many researches similar effects were observed after application of the stressful stimuli, such as prolonged immobilization and forced swimming stress.[16,17] Mingoti *et al*.,[17] in a study, demonstrated that a time period of 15 days forced swimming stress applied to adult male rats did not impair fertility, but significantly decreased spermatid production. They observed no changes in body, testicular, seminal vesicle, ventral prostate weights and histological features of the testes of stressed male.[17] Complete sequence of spermatogenesis constitutes 14 cycles, which lasts 48-53 days in the rat.[17]

In this sequel, we designed the hypothesis according to spermatogenesis period i.e. 48-50 days in the rat.[18] The purpose of the present study was to determine whether 50 days forced swimming stress applied to adult male rats affects count, motility and fertilization capacity of sperm.

## MATERIALS AND METHODS

### Animals

This experimental study was performed in the Cell Culture Laboratory of Ahwaz Jondishapour University of Medical Sciences (AJUMS) from September 2008 to April 2009. A total of 30 adult male Wistar rats, 3 months of age, weighing 210 ± 10.6 g were purchased from the Laboratory Animals Care and Breeding Center of Ahwaz Jondishapour University of Medical Sciences, Ahwaz, Iran.

The fertilizing ability of male rats was proven at the beginning of the experiment by selecting post first wave of spermatogenesis[19] that mate and observed positive pregnancy. All rats were randomly divided into two equal groups (n = 15): (1) control and (2) experimental groups. All animals were housed individually per cage under a 12-h light/dark cycle, 20 ± 2°C temperature and 60-65% humidity-controlled room with food and water *ad libitum*. All procedures were approved by international guidelines and by the Institute Research Ethics and Animal Care and Use Committee of Ahwaz Jondishapour University of Medical Sciences. Every effort was made to minimize the number of animals used and their suffering.

### Experimental design

Animals in the control group remained undisturbed in their cages but the experimental group was submitted to force swimming for 3 min in water at 32°C daily for 50 days, as previously described by Mingoti *et al*.[17] Stress was assessed by the hot-plate test after the last stressing session. In the hot-plate, the plate temperature was 52°C and the maximal cut-off time was 60 s. The latency time for hind paw licking after exposure to the hot-plate surface was measured and the increase in relation to control was considered to be an index of the antinociceptive effect.

### Sperm analysis

Sperm count and motility of the two study groups were determined using a Makler chamber. All counts were performed at 37°C in T6 media. The sperm motility was assessed and classified as progressive and non-progressive. Initial sperm motility was manually assessed by a single individual in duplicate for each sample by evaluating 100 sperms. Total motility was defined as any movement of the sperm head and progressive motility was defined as the count of those spermatozoa that moved in a forward direction.

### Oocyte collection

Adult female wistar rats that were between 10 and 12 weeks old were administered intraperitoneally with 10 IU pregnant mare serum gonadotropin (PMG) for superovulation. This was followed 46-48 h later by the intraperitoneal administration of 10 IU human chorionic gonadotropin (hCG). Rats were killed 12-14 h after hCG injection by cervical dislocation method. After disinfection with 70% alcohol and opening the abdomen wall, the Y-shaped uterus, ovaries and oviducts were identified. The oviducts were excised as follows: Clamping cornuas, dissecting the peritoneum and fat between ovary and tube and then cutting the fallopian tube from the proximal end and cumulus–oocyte complexes were collected in KSOM medium. The granulosa cells of oocytes were removed by pipetting in KSOM medium containing 80 IU/ml hyaluronidase and mature oocytes obtained and randomly divided into two groups.[19]

### *In vitro* fertilization

*In vitro* fertilization was carried out in drops of KSOM medium plus 5 mg/ml bovine aerum albumin (BSA) under mineral oil. A pre-incubated capacitated sperm (motile and non-motile) suspension of different groups as mentioned above was gently added to the freshly collected ova, which divided into two groups to give a final motile sperm concentration of 100,000/ml. The combined sperm–oocyte suspension was incubated for 4-6 h under a condition of 5% CO_2_ and 37°C temperature. The ova were then washed through several changes of medium and finally incubated in drops of T6 + 5 mg/ml BSA under mineral oil. Fertilization was assessed by recording the number of two-cell embryos 24-26 h after completion of fertilization *in vitro*.[20]

### Statistical analysis

Data are reported as mean ± SD and percentage. The statistical significance of difference between the control and experimental groups was determined by the unpaired *t*-test. Differences between the means were considered to be significant when *P* < 0.05 was achieved.

## RESULTS

The number of sperm and percentage of spermatozoa showing progressive motility, non-progressive and fertilization capacity are expressed in Table 1. The sperm concentration of male rats in the control and experimental groups were 60.8 ± 9.3 10^6^/ml and 20.4 ± 5.3 10^6^/ml, respectively [Table 1]. There was statistically difference (*P* < 0.05) between the two groups in terms of sperm concentration (*P* < 0.05). The percentage of sperm with progressive motility was 53.25 ± 3.97 in the control group and 23.87 ± 2.58 in the experimental group. The difference was significant (*P* < 0.05). The percentage of non-progressive sperm motility significantly increased (*P* < 0.05) in the experimental group [Table 1]. The fertilization capacities of male rats were 20 and 60.7 in the control and experimental groups, respectively [Table 1]. The difference in fertilization capacities between the two groups of the study was significant (*P* < 0.05). The same results were obtained in case of fertility (*P* < 0.05). Stress caused by forced swimming was observed by a significant increase in the latency of the pain response in the hot-plate test (*P* < 0.05).

**Table 1:** Effect of 50 days of forced stress on count, motility and fertilization capacity of sperm in adult male rats

Study group variables	Latency time for hind paw licking (s)	Sperm concentration (10^6^/ml)	Progressive sperm motility (%) (mean ± SD)	Non-progressive sperm motility (%) (mean ± SD)	Fertilization capacity (%)
Control group	15.23 ± 2.28	60.8 ± 9.3	53.25 ± 3.97	25.62 ± 3.50	60.7
Experimental group	27.81 ± 1.82	20.4 ± 5.3	23.87 ± 2.58	50.26 ± 3.19	20.0
*P*-value	0.04	0.001	0.005	0.00	0.007

## DISCUSSION

Complete sequence of spermatogenesis constitutes 48-53 days in the rat.[17] The present data demonstrated that sperm count significantly decreased after 50 days of forced swimming. In agreement with this data, Mingoti *et al*.[17] claimed that the normal fertility exhibited by stressed males may be explained by the fact that forced swimming lasted 15 days, a period shorter than 48 to 53 days, which may not have affected the spermatogenesis significantly. Thus, the present data suggest that the effects of stress on fertility should be better assessed after the period of time necessary to complete an entire cycle of the spermatogenesis, between 48 and 53 days in the rat.[17]

Literature showed that spermatogenesis has been inhibited in response to various stressors.[15,16,21] This study also observed that the progressive motility of male rat sperm significantly decreased after 50 days of forced swimming, which may highlight the harmful effect of forced swimming on motility of rat sperm. The reduced fertilization capacity of stressed male is probably due to a decrease in the number and motility of sperm. It is known that the production of spermatozoa able to fertilize and develop a normal progeny results from normal sperm maturation in the epididymis. The composition of the internal epididymal milieu, responsible for sperm maturation, is under androgen control.[22]

In rats, an androgen-binding protein secreted by Sertoli cells into the lumen of seminiferous tubules under follicle-stimulating hormone stimulation is transported to the epididymis, where it accumulates at concentrations higher than those found in the testes. This leads to a high local concentration of androgens, essential for maturation of epididymal spermatozoa.[16] A previous study reported that adult male rats exposed to prolonged immobilization exhibit a decrease in spermatid production and sperm density,[23] in addition to lower plasma testosterone concentration and a small but significant reduction in the amounts of Sertoli cells per seminiferous tubule cross.[16]

## CONCLUSION

The present data demonstrate that forced swimming after the period of time necessary to complete an entire cycle of the spermatogenesis, between 48 and 53 days, when applied to adult male rats, the number of sperm, fertilization capacity as well as motility of sperm will significantly decrease. This result may raise the attention to effect of stress in humans in terms of sports training and stress from day to day life.
